# Wet Dressings in Surgery*Presented in Section on Surgery and Anatomy, at the Forty-ninth Annual Meeting of the American Medical Association, held at Denver, Colo., June 7-10, 1898.

**Published:** 1898-09

**Authors:** Thomas Osmond Summers

**Affiliations:** M. A., M. D., F. S. Sc., London; Professor of Anatomy and Orthœpedic Surgery in the St. Louis College of Physicians and Surgeons, St. Louis, Mo.


### WET DRESSINGS IN SURGERY.*

BY THOMAS OSMOND SUMMERS, M. A., M. D., F. S. Sc., LONDON.

Professor of Anatomy and Orthœpedic Surgery in the St. Louis College of Physicians and Surgeons, St. Louis, Mo.

This may well be called the Dressing Period in the evolution of surgery. Time was when the scapel alone was emblazoned on the escutcheon of the surgeon and with the skillful incision his responsibility ended, nor did the dignity of his office admit of his performing what were then held as the minor and menial offices of after-treatment, which then was supposed to cover everything that followed upon the first brilliant sweep of the surgeon's glittering steel. There are, no doubt, some who hear me to-day who remember the dramatic toss of the knife behind him of the elder Gross when completing his incision and the autocratic delivery of the case to his assistants for the dressing and treatment of the wound.

Even to this day the red, white and blue stripes of the barber pole tell of surgery's humble origin and the bandagers and bone-setters still roam through the villages of England and the barber-surgeons still apply the leech and cup for the more dignified practitioner. It remained for Sir Joseph Lister to break the spell of this *otium cum dignitate*, which was the bane of all surgical progress, and teach the autocrats of the scapel that surgery meant much more to the organism than the mere solution of continuity along anatomical lines—that this indeed was the *avant courier* of the real principle from which all the almost miraculous achievements of modern surgery had been evolved. It was my privilige to be present at the presentation to the British Association, at Edinburgh, in 1875, by Professor Lister for the first time of a clinical demonstration of his mode of surgical dressing which opened to surgery new worlds to conquer. The case was one of ligation of the external iliac, and the elaborate dressing being removed proved the triumph of his principle, though his venerable colleague, Professor Spence, almost on the verge of eternity, threw a well-poised Parthian lance at the rising genius of modern surgery. Since that day every operation, however simple in itself, has been one in which the surgeon "earned his bread by the sweat of his brow." No turning over the case to the unwashed student, for he whose records of success are proclaimed to-day is the operator who leaves not his patient until the last jot and tittle of aseptic dressing has been fulfilled, for so exacting is this principle that *falsus in uno, falsus in omno* is the inflexible law of its operation.

As a matter of course, with the increase of such labor necessary as it has proved to be to insure surgical success, the in-

*Presented in Section on Surgery and Anatomy, at the Forty-ninth Annual Meeting of the American Medical Association, held at Denver, Colo., June 7-10, 1898.

genuity of man was set to work at once to simplify the methods without impairing the efficiency of aseptic surgery. So active has been the work in this field that apparatus of every device and design has been offered to the profession until its name is legion, and of making of dressings, as the patriarch said of books, "there is no end." As in everything else, the element of "faddism" or surgical "fashion" has been dominant even in this unesthetic field, and the very men who pretend above all others to condemn the follies of feminine fashion are themselves afraid to say their soul is their own when it comes to operating and dressing of wounds before their lynx-eyed professional rivals. For years past, for example, it had been almost as much as a surgeon's name was worth to apply oleaginous preparations to surface leisons, although the very first treatment of wounds of which we read recommends the "pouring of oil," and in the case of the good Samaritan received endorsement of the Great Physician himself.

There are some things, however, that fashion can not forbid, and this is one of them—like Banquo's ghost, "it will not down." Long before science had thrown its searchlights over the dark field of biogenesis, experience had taught the steel-clad warrior the virtues of Gilead's balm, and from the shades of Olivet where fell the tears of Him who came for the healing of the nations, man had learned to gather the oil for his wounded body. Since the discovery of the bacteriologic processes of infection in open wounds, there has been a gradually growing tendency to return to the ancient remedial agents which experience dogmatically taught were rationally indicated. In the last edition of that eminently practical work upon surgery by Wyeth, of New York, we find this positive and significant utterance upon the use of oil and balsam, the first surgical dressings known to humanity: "I know of nothing equal to this valuable preparation. The oil acts in a twofold way—the surface of the wound is moistened by it, while the liquid excretion from the wounded surface is carried off in the dressing by capillary attraction. The removal of moisture cripples the proliferation of the bacteria and in this way aids in antisepsis."

In the process of repair, all the structural elements must be supplied from beneath the surface of the lesions, so that the constructive metabolism would not be injured by the mechanical interference of the oil globules, which, as intimated in the quotation just made, "would cripple the proliferation of bacteria" which comes from without. There are many wet dressings which if frequently renewed show excellent results, but there are few cases which come before us in which disturbance of dressing does not do mechanical injury to the process of repair, besides exposing the wound to the entrance of pyogenic and other cocci while the dressing is being changed, while the retention of material which has expended its aseptic influence is a constant menace to the integrity of the organism at large. Lister was working to the overcoming of these difficulties when he devised his "paste" dressing, which, however, failed to meet the desired ends in many cases. The use of animal and vegetable oils is also open to the objection offered to solutions in dressings—the necessity of changing the dressing too often—but for a different reason, the tendency of the oils to become rancid, and this applies also to the keeping of such dressings prepared for use. Wyeth recommends in his oil and balsam dressing the sterilization of the oil before using, but admits that this is often impracticable and recommends in this case the use of plain cold castor oil of the shops.

It is therefore clear that to carry out the idea of a practical dressing it must be:

1. Antiseptic. This applies not only to the effect of the application to the part affected, but to the corporate substance itself, thus insuring it against auto-infection before applying.

2. Permanent. This is necessary in order to avoid too frequent removals as well as to preserve itself from deterioration.

3. Non-irritating. There is nothing more delicate, more easily disturbed than the formative principles of tissue, so that care must be taken in the dressing of all lesions of surface lest the agents used should arrest the tender process of repair, as well as protect it from the invasion of destructive germs.

4. Constructive. While the majority of leisons, especially those of traumatic orign, if not interfered with by destructive germs will heal rapidly of themselves, there are very many which require not only this negative condition, but also a positive stimulation of function in the constructive elements of the part. Stimulation of cell growth, however, must not go to the extent of irritation, in which case there will be destruction instead of construction of tissue.

The fulfillment of these conditions has been the aim of the surgical pharmacist from the time when the first coccus wriggled across the field of the microscope and gave its first exhibition to the scientific investigator of its dance of death within the organism of man. But amid all this elaboration of apparatus it was to Sir Astley Cooper after all that the credit is due, for his foreseeing therapy leaped over, as it were, the dark chasm which separated the triumphs of his surgical genius from the science-illumined land of modern surgical pathology. It was he who, without the knowledge of the bacteriologic factor in the great problem of surgical treatment, by the intuition of genius gave to us the essential principles of external dressing for surface lesions. His formula, however, was open to the objection of violating one of the conditions herein laid down—that of permanence—in that lard was used instead of petrolatum, which has been since discovered and is now substituted in the preparation known as Unguentine, which is an ideal formula constructed along the lines of that suggested by Sir Astley Cooper, but altered to the conditions of modern aseptic surgery. The irritating effects of the ordinary alum has also in some way been obviated, furnishing thus a typical dressing for surface lesions. For internal lesions that are to be immediately and permanently closed beneath the sutured integument there are many valuable aseptic liquid preparations which we prefer to the too indiscriminate use of iodoform, aristol *et id omne genus*, but we are free to admit that for all external dressings we have found the highest fulfillment of modern aseptic or antiseptic surgery in the preparation just mentioned.

I am not sure whether this is a proprietary preparation or not, but this I do know, its formula is an ideal one and its results are certainly very satisfactory. It is about time we were looking around after labor-saving methods when we have to employ at the simplest incisive operations an extra attendant to wipe the sweat from our brows as the houri fans the Sultan's heated cheek—though our attendants are not all houris, nor are our cheeks fired with the congestion of a lazy passion. We are glad to see this unholy war against oleaginous applications coming to an end, just as we should be also glad to see the phlebotomy pendulum point to the nadir. In surgical politics I am a middle of the road man—*In medio tutissimus ibis.—The Journal of The American Medical Association.*

